# Investigation of ring, airjet and rotor spun yarn structures on the fragmented fibers (microplastics) released from polyester textiles during laundering

**DOI:** 10.1177/00405175231191785

**Published:** 2023-08-09

**Authors:** Abdul Jabbar, Muhammad Tausif

**Affiliations:** 1 School of Design, University of Leeds, UK; 2 Department of Textile Engineering, 66726 National Textile University, Pakistan

**Keywords:** Fragmented fibers, microplastics, yarn structure, polyester fibers, fiber damage

## Abstract

The release of fragmented fibers (FFs), including microplastics from textiles, during their service life is considered an established source of environmental pollution. The yarn structure is identified to affect the amount and length distribution profile of shed FFs from textiles. In the present work, the impact of yarn structures spun from 100% polyester staple fibers, using commercially relevant spun yarn technologies in the textile industry, on the release of FFs from textiles is studied. The bespoke woven fabric samples produced from three types of spun yarns, which include ring, airjet (air vortex) and rotor yarns, were subjected to an accelerated washing process, for up to five washes, to quantify shed FFs and their length distribution profile. The morphological shapes of FF ends associated with the nature of fiber damage were also investigated. The results demonstrated that airjet and rotor yarn structures had released 28% and 33% less mass of FFs, respectively, as compared to the ring yarn structure during the whole washing process. The length distribution profile identified that the ring yarn structure shed longer length FFs as compared to both airjet and rotor ones. The damaged ends highlight the importance of textile manufacturing processes on the generation of FFs. The results of this study give a better understanding of the yarn structural effect of commercially relevant technologies on shedding of FFs, which are released as a pollutant to the environment.

The existence of microplastics is one of the major environmental challenges. Fibrous microplastics are the dominant type of microplastics found in the aquatic and terrestrial environment and textiles are reported to be a major source of fibrous microplastics.^1,2^ Both natural and synthetic textiles, manufactured from staple and filament yarns, release fragmented fibers (FFs; including fibrous microplastics, usually <5 mm in length)^3^ during their entire life cycle starting from production to service life to end of life disposal.^4^ Previous studies highlight that a large segment of FFs observed in the environment are released during the laundering of textiles.^1,5–7^ Fast fashion trends and the growing world population are mainly responsible for the increasing production and consumption of fibers and textiles.^8^ Synthetic fibers account for approximately 60% of the global consumption of textiles fibers.^9^ The share of polyester (PET) fiber alone accounts for more than 54% in the global textile industry,^10^ which is expected to grow in the coming years.^11^ Therefore, PET is considered an important textile fiber, and most of the previous laundering studies primarily focus on PET fabrics.

The abrasive wear and fiber damage during manufacture, use and laundering result in the generation and release of FFs from textiles.^8^ Different parameters responsible for the generation and release of FFs from textiles include the physicochemical properties of textile fibers and their morphology, yarn type and structure, fabric type and geometry and textile processing history.^8^ Raja Balasaraswathi and Rathinamoorthy^12^ reported the effect of the knitted fabric structure and fabric structural parameters on FF shedding from 100% PET textiles. The interlock knitted structure resulted in more shedding of FFs during laundering as compared to 1 × 1 rib and single jersey structures. Moreover, higher stitch density, higher tightness factor, lower loop length and lower fabric mass areal density resulted in reduced FF shedding. A study on the impact of key yarn structures and material composition on the release of FFs during laundry pointed out that textiles manufactured from both multifilament and staple spun yarns release FFs.^3^ Another study disclosed similar results.^1^ De Falco et al.^13^ reported the lowest release of FFs from textiles with a very compact woven structure and highly twisted continuous filament yarns compared to that of an open structure. Berruezo et al.^14^ presented the influence of a weave pattern with a high interlacing coefficient and yarn densities on the lower release of FFs from textiles. Textile processing history also influences the release of FFs from textiles as, reported by a few studies.^15–18^ Cai et al.^15^ explored the presence of FFs (microplastic fibers) in various intermediate fiber products, and for a number of different finished PET textiles. However, the effect of each textile manufacturing process (which generally includes spinning, weaving/knitting, dyeing and finishing) on the formation and release of FFs was not investigated.

The yarn structure (i.e. the way constituent fibers are geometrically arranged and bound in the yarn body) not only affects its properties but also the generation and release of FFs from textiles, as disclosed in a recent study.^10^ Staple spun yarns are dominantly used in the manufacturing of apparels and home textiles, accounting for 45% of global yarn production.^19^ The geometrical arrangement of fibers in the yarn depends on the spun yarn manufacturing system. Among different spinning systems, ring spinning is the oldest and most widely used technology in the spun yarn industry due to yarn quality attributes being acceptable to a wide range of textile applications and its versatility to spin a wide range of yarn counts.^20^ However, the low productivity and high production cost make this system less sustainable as compared to alternative yarn spinning systems. Rotor spinning and vortex (airjet) spinning have gained wide commercial acceptance worldwide among different alternative yarn spinning systems.^21^ The high productivity and low production cost make these alternative systems more sustainable compared with ring spinning, but each system has its own limitations in terms of end use and the limited yarn linear density (count) range.^22^ Despite that, the yarns of both systems have gained application in certain apparel and home textile end uses due to the higher productivity and unique properties they offer, that is, low hairiness and better evenness compared with ring spun yarn.^23^

A literature survey shows that there is a paucity of work on the effect of yarn structures of commercially relevant spun yarn technologies in the textile industry on the release of FFs from finished textiles. According to the authors’ knowledge, there are two studies that discovered the effect of ring, rotor and airjet yarn manufacturing processes on the formation and presence of FFs (microplastic fibers) in PET yarns.^15,24^ However, the quantity of FFs extracted in the laboratory from undyed yarns was much higher as compared to dyed yarns. For example, 2244 ± 60 FFs/g were extracted from undyed rotor yarn^24^ as compared to 884 ± 154 FFs/g extracted from the dye effluent of dyed rotor yarn and 455 ± 58 FFs/g extracted from the same dyed yarn during the subsequent first extraction cycle in the laboratory.^15^ These outcomes highlight the influence of wet processing on the release of pre-existing FFs from preceding manufacturing processes (spinning, weaving/knitting) and demonstrate that the results of dyed yarns and fabrics can be different from those of the midway products (i.e. undyed yarns and fabrics) due to the intermediate manufacturing steps, especially processing under wet conditions. Therefore, the objective of this study was to understand the effect of ring, airjet and rotor spun yarn structures on the release of FFs from dyed PET fabrics during washing. Bespoke yarns were converted into woven fabrics and subsequently dyed under controlled conditions for a realistic comparison. The extracted FFs, after washing, were characterized in terms of gravimetric mass, length distribution profile and count. The morphological shapes of FF ends associated with the nature of fiber damage were also investigated. The results of this study will not only help to give an insight into the effect of the commonly used spun yarn structures on the release of FFs from textiles, but also provide understanding of yarn structural choices to mitigate the generation of FFs released during use and service.

## Materials and methods

### Yarn and fabric production

**Figure 1.**
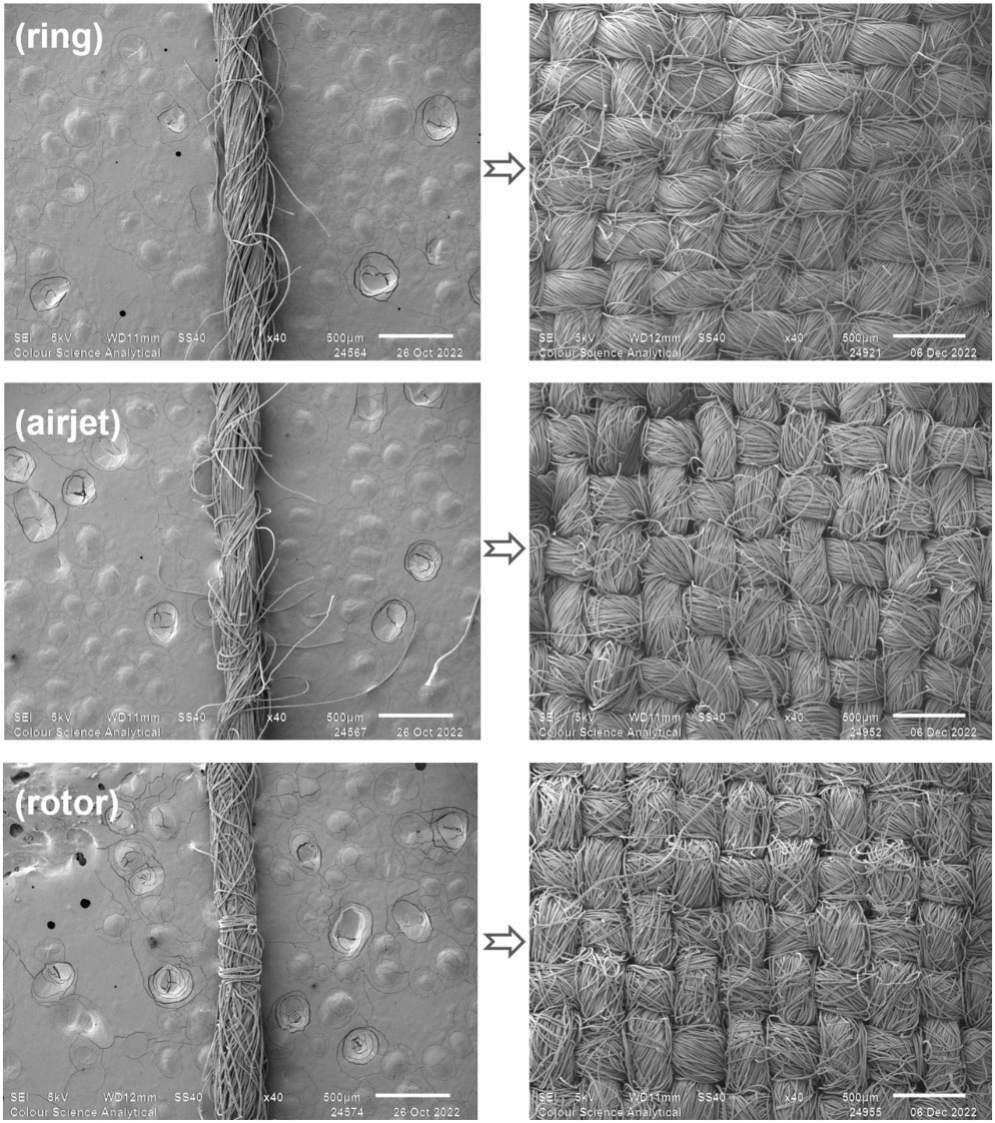
Scanning electron microscopy images of 100% polyester staple spun yarns and their corresponding dyed fabrics.

PET staple fibers (specifications given in Table 1) were employed to produce conventional ring, airjet (vortex) and rotor spun yarns. Ring, airjet and rotor spinning are the commercially relevant short-staple fiber spinning systems and the properties of the resultant yarns vary due to differences in the yarn formation principles. A detailed description of the yarn formation principles of these spinning systems is reported elsewhere.^25^ The raw virgin PET fibers were processed through a blow room line (Rieter B34, A21, A79, A21), carding machine (Rieter C75), breaker drawing frame (Toyoda DYH 500C), finisher drawing frame (Rieter RSB D40), roving frame (Toyota FL-16) and ring frame (Toyota RY-5) to produce conventional ring spun yarns. The finished drawn sliver from the above preparatory process was processed through a rotor spinning machine (Schlafhorst SE-8 Autocoro) and a Murata vortex machine (vortex 870) separately to produce rotor and airjet yarns, respectively. All yarns were produced with a nominal linear density of 29.53 tex (20.0 Ne). The nominal twist level in conventional ring spun yarn was kept at 7.04 turns.cm^−1^ (17.88 turns.inch^−1^). However, the true twist in airjet and rotor yarns cannot be measured due to different yarn formation principles. The structures of yarns spun using ring, airjet and rotor spinning systems and their corresponding woven fabrics are shown in Figure 1. The properties of the developed yarns are given in Table 2. It can be seen in Figure 1 that the ring yarn shows a uniform helical arrangement of fibers in the yarn body, while the airjet yarn shows a parallel arrangement of fibers in the core bound together by wrapper fibers. The rotor yarn shows bellyband and wild wrapper fibers on its surface. This yarn usually has densely packed fibers in the core.^25^ The ring fabric shows higher fiber surface fuzziness as compared to the airjet and rotor fabrics. The yarns were converted into plain-woven fabrics with thread densities of 23.62 threads cm^−1^ (60 threads inch^−1^) in the warp direction and 22.05 threads cm^−1^ (56 threads inch^−1^) in the weft direction on a laboratory-scale rapier-weaving loom (CCI Tech Incorporated Taiwan). The warp yarns were treated (sized) with polyvinyl alcohol (PVA) solution before weaving to improve the weavability of the yarn by improving the strength and reducing the metal–yarn friction. The fabric samples were named as ring, airjet and rotor based on the yarn type.

**Table 1.** Properties of polyester used in the study

Sr. no.	Parameters	Values
1	Fiber cut length, mm	38
2	Fineness, denier (tex)	1.2
3	Tenacity, cN/tex	60.2
4	Tenacity at 10% elongation, cN/tex	46.7
5	Breaking elongation, %	23.8
6	No. of crimp/25mm	10.4

**Table 2.** Properties of spun yarns

Sr. no.	Spun yarn	Actual count (tex)	Unevenness (%)	Total imperfections/km	Hairiness index (−)	Tenacity (cN/tex)	Breaking elongation (%)	Zweigle hairiness, S3
1	Ring	28.56±0.39	10.85 ± 0.63	57.6 ± 19.72	6.29 ± 0.15	29.28 ± 2.82	22.22 ± 1.16	1356 ± 185
2	Airjet	29.02 ± 0.17	9.45 ± 0.11	26.5 ± 7.37	3.79 ± 0.03	27.36 ± 2.10	21.16 ± 1.16	2.20 ± 2.56
3	Rotor	28.84 ± 0.11	9.01 ± 0.46	50.9 ± 10.74	5.42 ± 0.04	21.74 ± 1.81	20.80 ± 1.41	415 ± 80.66

### Fabric dyeing

The fabric samples were pre-washed with 5.0 g.l^−1^ AATCC reference detergent at 70°C for 45 min, maintaining a liquor ratio of 10:1 in a laboratory-scale Jet machine (Japan) followed by rinsing with tap water. The washed fabric samples were dyed with Foron® Navy Blue (Archroma, Singapore) disperse dye. The dyeing process was carried out using 5% omf of dye and 0.5 g.l^−1^ dispersing agent at 130°C for 60 min maintaining a liquor ratio of 20:1 in the dye bath. The pH of the dyeing bath was adjusted to 4–4.5 using acetic acid. The dye bath solution was cooled down and drained, followed by a hot washing of samples at 70°C for 15 min. Finally, the samples were rinsed thoroughly with tap water, neutralized and dried at room temperature.

### Sample preparation, washing and FF mass measurements

The standard test method AATCC TM212-2021 was followed for preparation and washing of fabric samples. Four specimens from each fabric sample were scissor cut into sizes of 200 × 340 mm^2^. The specimens were folded under (to the back) 50 mm from each edge and sewn close to the inner edge using lockstitch (stitch type 301, 2.5 mm per stitch) and Coats Astra (Tkt 120, 13ANT) 27 tex 100% staple spun PET thread of light green color. The sewing thread color was intentionally kept different from the colors of the textile samples to differentiate any release of FFs from the sewing thread.^26^ The final sizes of the specimens after sewing were approximately 100 × 240 mm^2^. The textile manufacturing processes involve fiber–fiber, fiber–metal and fiber–water interactions, which are known to cause fiber damage, leading to a higher amount of FF generation in the first cycle.^26^ Hence, the specimens of all samples were subjected to a pre-washing step, without using any steel balls, to collect FFs associated with textile manufacturing as well as to remove any excess dye, dust and dirt particles. Subsequently, the fabric specimens were washed for up to five washing cycles using standardized laboratory laundry equipment (GyroWash, James Heal) and adding 50 steel balls (6 mm diameter) per canister. The addition of balls enhances the mechanical stresses during the washing process equivalent to five domestic washes in an industrial laundry in relation to color fastness.^27^ The pre-washing and five washing cycles were carried out by putting the fabric specimens in stainless-steel canisters with 360 ml of detergent solution (2.5 g detergent per 1000 ml distilled water) per canister at 40°C for 45 min at 40 rpm. The canisters were thoroughly washed with distilled water before each washing cycle. AATCC high efficiency standard reference liquid detergent without optical brightener was used for gyro washing. Each washing cycle was conducted with four replicate specimens for all samples to compute average values.

The fabric samples after each cycle were rinsed three times using distilled water separately, and the effluent was recovered for subsequent filtration. A pair of tweezers was used to remove excess water from the textile specimens, and the detergent foam was removed by rinsing the specimens in distilled water. The open mesh, canister, beakers and tweezers were rinsed three times. All recovered effluent from each textile specimen, open mesh, canister, beaker and pair of tweezers was then collected in one beaker for subsequent filtration. The beaker and glass funnel were also rinsed three times, and all the recovered effluent was filtered using a binder-free glass fiber filter of 1.6 µm mean pore size and 47 mm diameter (Merck Millipore Ltd, Ireland). The filters were weighed before filtration using a precision balance (Mettler Toledo AE160, resolution of 0.00001 grams). After filtration, the filters were placed in a fan oven overnight at 50°C, kept in a desiccator for 1 hour and re-weighed to determine the increase in filter mass, which corresponds to the amount of released FF from the textile specimens. The textile specimens after each washing cycle were left to dry in a fan oven overnight at 50°C and then used again for the next washing cycle.

### Quantification and length distribution profile of FFs

The quantification and length distribution measurement were performed on a Diamlength instrument (Cottonscope Pty Ltd, Australia) using the automatic image processing of digitized real-time images of the water-immersed FFs. The glass filter, with the FF mass over its surface, was submerged in approximately 1 l of distilled water in the glass beaker for 5 min. The water was stirred using a glass rod for a few seconds to loosen and disperse the FFs. This process successfully released the FFs from the surface of the glass filters. As the textiles were dyed blue in color, the successful removal of FFs was assumed when no blue colored mass was observed over the surface of the glass filters. However, many glass fibers were also detached from the glass filter during stirring and dispersed in the water, and were subsequently rejected from measurement due to their different material density and refractive index, by employing the opacity parameter of the Diamlength instrument. Three specimens from each sample were used for quantification to calculate the average value.

### Scanning electron microscopy

The microscopy of the manufactured yarns and the corresponding fabrics was done using a scanning electron microscope (Jeol JSM-6610, Japan) to understand the differences in their surface morphology and physical structure. All samples were sputter-coated with an approximately 30 nm gold layer by using a sputter coater (Q150RS, Quorum Technologies). The damaged ends of FFs collected on the glass filters from the pre-wash and the fifth wash of ring, airjet and rotor samples were characterized using the same scanning electron microscopy (SEM) to elucidate the mechanism of fiber damage. Twenty fiber ends were randomly imaged from each sample after the pre-wash and fifth wash to estimate the details of the broken fiber end morphologies. In addition, SEM was also used to evaluate the surface of the textile samples before washing and after the fifth wash in order to identify the fiber surface damage/wear.

### Statistics and quality control

Tukey’s comparison method, using one-way analysis of variance (ANOVA), was employed to compare the statistical significance of the effect of the yarn structure on the mass and number of FFs of the ring spun fabric to those of the airjet and rotor fabrics. The order of washing experiments was randomized to minimize the chances of systematic error. Two blank runs containing only detergent solution in canisters were randomly inserted into a washing cycle, with a total of 14 blank runs of all washing experiments, to determine the level of contamination during washing and filtration. During the experiments, protective nitrile gloves and a white laboratory coat were worn. A very small number of contaminants with an average mass of 0.08 ± 0.05 mgwere observed, while the average number of contaminant fibers/particles manually counted with the help of a light microscope from 14 blank runs was 110 ± 32.

## Results and discussion

### FF mass and length distribution

**Figure 2.**
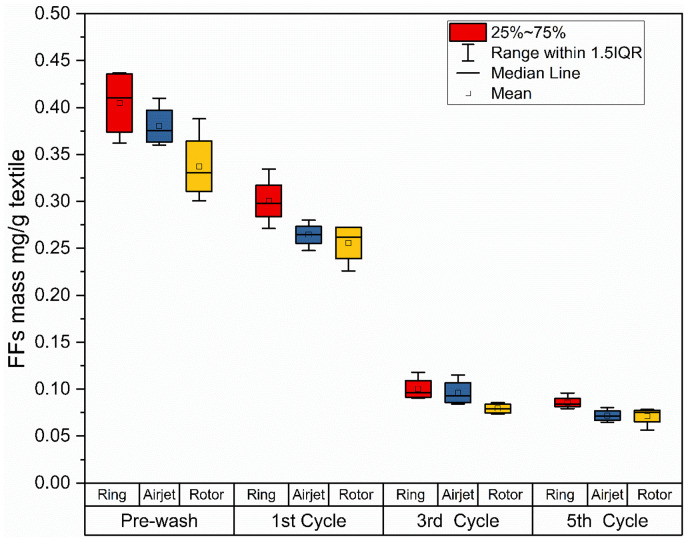
Fragmented fiber (FF) mass collected from wash effluent after the pre-wash, first, third and fifth laundry cycles. The plot of data is based on quadruplicate experiments.

The mass of FFs released from the textile samples and collected after the pre-wash, first, third and fifth wash cycles is plotted in Figure 2. A higher release of FF mass is noted during the pre-wash for each type of fabric despite adding no steel balls, as compared to the first wash cycle, which continues to decrease in subsequent cycles. It has been reported in multiple research studies that the release of FFs is decreased with the increasing number of washing cycles, reaching a plateau at the fifth cycle^3,7,10,28,29^; therefore, the data from the pre-wash and every alternate wash cycle until the fifth wash cycle were considered in this study. As per Figure 2, ring spun fabric generally releases a higher mass of FFs as compared to airjet and rotor fabrics during each washing cycle; however, this difference among the extracted mass of samples was found to be reduced from the third wash cycle onwards. In terms of accumulative mass of FFs collected from all washing cycles, the airjet and rotor samples released 28% (average FF mass 0.67 mg/g textile) and 33% (average FF mass 0.63 mg/g textile) less mass, respectively, as compared to the ring samples (average FF mass 0.94 mg/g textile). This lower release of FF mass from the airjet and rotor fabrics was also found to be statistically significant at the 95% confidence level for airjet (*p*-value = 0.000) and rotor yarn fabrics (*p*-value = 0.000) as compared to the ring yarn fabric. However, the FF mass from airjet samples was found to be statistically insignificant at the 95% confidence level (*p*-value = 0.154) as compared to the rotor samples. Despite adding no steel balls during the pre-wash step, where the objective was to stimulate the release of FFs associated with textile manufacturing processes, the release of FF mass was noted to be higher, for each textile sample, as compared to subsequent wash cycles where steel balls were added to accelerate the laundry process. This finding indicates the significance of textile manufacturing processes on the generation of FFs, which has already been disclosed in earlier studies.^15,24^

**Figure 3.**
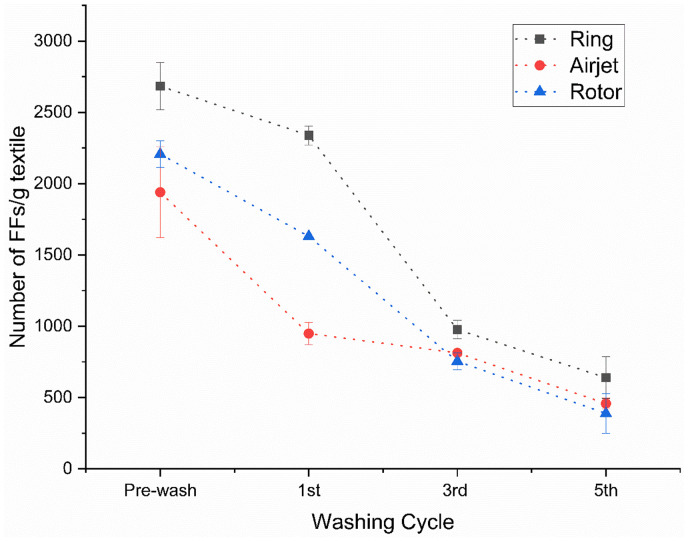
The number of fragmented fibers (FFs) released from textiles during laundry cycles. The plot of data is based on triplicate experiments.

**Figure 4.**
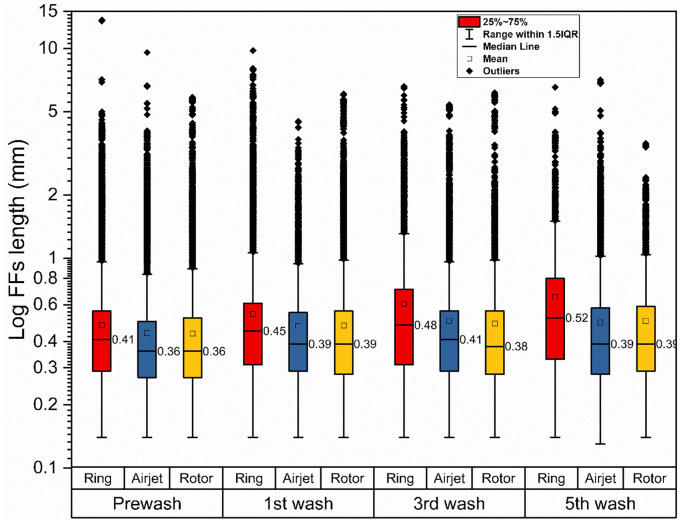
Fragmented fiber (FF) length distribution profile after the pre-wash, first, third and fifth laundry cycles. The data plotted is the sum of three experimental replicates.

**Figure 5.**
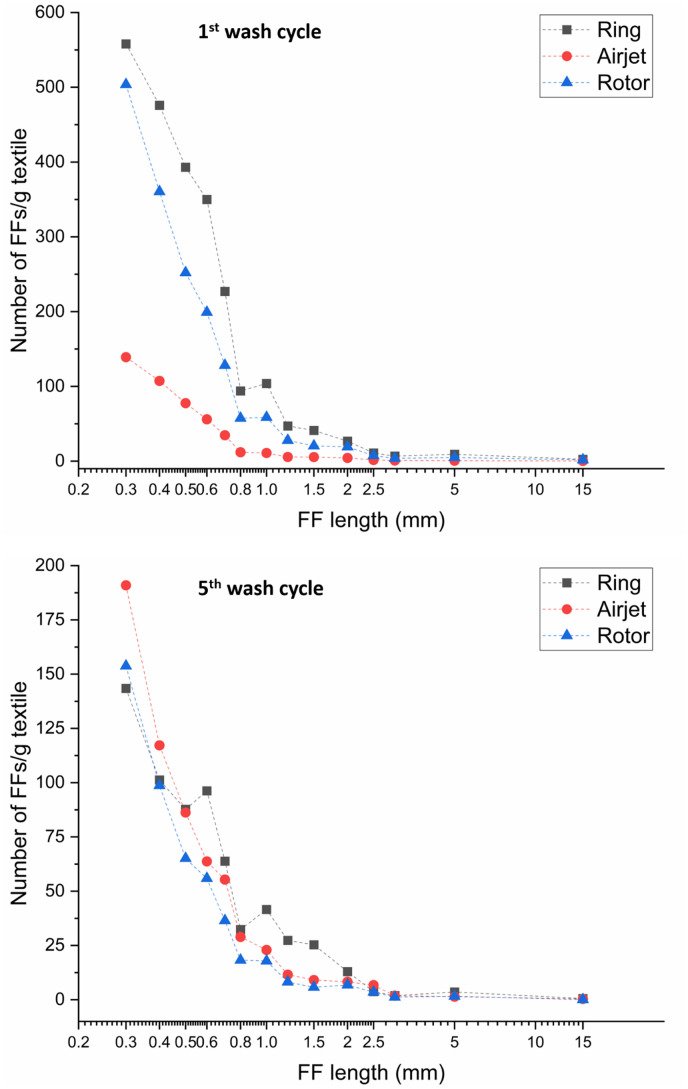
Fragmented fiber (FF) length distribution profiles against FFs per gram of textile for the first and fifth cycles.

The number of FFs released from the ring, airjet and rotor samples shows a decreasing trend similar to the decrease in gravimetric FF mass during repeated washes. As shown in Figure 3, the FF count was higher during the pre-wash for all samples, and decreases gradually in subsequent washing cycles. One important aspect, common in relevant studies, is the decrease in the release of FFs after repeated washing cycles reaching a plateau near the fifth wash cycle.^28,29^ The same attribute was observed in the current study. By calculating the total amount of FF released from all washing cycles, the airjet and rotor samples were observed to release on average 35% (4158 FFs/g textile) and 25% (4979 FFs/g textile) fewer FFs per gram of textile, respectively, as compared to the ring samples (6638 FFs/g textile). The Tukey’s comparison showed a statistically significant difference at the 95% confidence level for the airjet (*p*-value = 0.001) and rotor samples (*p*-value = 0.007) as compared to the ring samples. The FF count from the airjet samples was also found to be statistically significant at the 95% confidence level (*p*-value = 0.008) as compared to the rotor samples. In terms of FF length, the length distribution data was plotted on a logarithmic scale to visualize the large differences in length, as shown in Figure 4. The number of FFs varied significantly among the washing cycles and the textile samples; however, the difference in length distribution of the collected FFs was noted to be relatively small. In general, all samples released shorter FFs during the pre-wash as compared to subsequent wash cycles as evident by the increase in median length over cycles, as shown in Figure 4. There was a substantial increase in the median length of FFs for the ring samples in subsequent washes. It was noted that the median length of the ring samples was increased from 0.41 mm for the pre-wash to 0.52 mm for the fifth wash. However, this increase in median length was comparatively less for the airjet and rotor samples, where an increase from 0.36 mm for the pre-wash to 0.39 mm for the fifth wash was observed for both sample types. These outcomes may be explained by the fact that the shortest FFs can easily come out and release the textile structure during the initial wash cycle and relatively longer and more entangled FFs need more time to be released from textiles during subsequent washes. It is interesting to note that the ring sample released comparatively longer length FFs during each wash cycle as compared to the airjet and rotor ones. This is also confirmed by plotting the length distribution profiles against FFs per gram after the first and fifth cycles, as presented in Figure 5. During the first and fifth washes, about 94% and 88% of released FFs per gram were less than 1 mm for the ring samples, respectively. However, both the rotor and airjet samples showed relatively greater values, releasing about 94% and 93% of FFs per gram, less than 1 mm, during the first and fifth cycles, respectively. These results may be explained by the differences in yarn structure and properties, especially the yarn hairiness (Table 2). The ring spun sample shows a higher hairiness value as compared to the airjet and rotor ones, which means that longer length fibers protrude from the ring yarn body. This may result in a greater probability of comparatively longer FF generation and release during subsequent textile manufacturing processes and washing cycles.

The staple spun yarn structure (i.e. the geometrical arrangement of fibers in the yarn) depends on the underlying principle of yarn formation. The structure not only affects the properties but also is likely to be an important parameter influencing the generation and release of FFs from textiles.^10^ Most of the previous washing studies have very limited control over the textile materials and manufacturing processes. However, in the current study, bespoke textiles were developed by employing PET fibers of the same specifications in a controlled pilot-scale textile manufacturing process to undertake a realistic comparison of the yarn structure on the release of FFs from textiles. Ring yarns can be spun to a broader yarn count range and are suitable for a wide range of textile applications due to the better handle properties of ring spun fabrics.^20^ In contrast to airjet and rotor yarns, the ring spun yarn is twisted and has a uniform helical arrangement of fibers with relatively uniform radial distribution of fibers in terms of fiber packing density.^30^ However, the ring yarn, spun with the same fiber specifications and yarn count, exhibits a higher hairiness value, as evident by the Zweigle hairiness S3 and Uster hairiness index values in Table 2. There is a correlation between higher hairiness, leading to ease of fuzz formation, and generation of more FFs.^31,32^ Both airjet and rotor samples release lower amounts of FF as compared to the ring one. As all material and process parameters were kept the same for all three textile samples, except the difference in the basic principle of yarn formation, the possible reason for these outcomes may be attributed to the difference in the yarn structures and properties. The airjet spun yarn has a unique fasciated structure where most of the fibers are in parallel configuration, forming the yarn core. These parallel oriented fibers are bound together by a small number of fibers wrapped around the yarn core (Figure 1). The airjet yarn also offers higher effective packing density as compared to conventional ring yarn spun with the same material and yarn count.^33^ This parallel configuration of fibers along with higher effective packing density of fibers in the yarn cross-section is the likely reason for the low release of FFs. Moreover, airjet yarn presents significantly less hairiness as compared to other yarns (Table 2) which may also contribute to less shedding of FFs. The rotor yarn structure offers twisted fibers in the core along with wild wrapper and bellyband fibers on the yarn surface (Figure 1). These twisted core fibers contribute to higher packing density fibers near the yarn center,^30^ which is likely to result in the release of fewer FFs during washing. The rotor spun yarn also demonstrates less hairiness than its ring spun counterpart, as presented in Table 2, which again can be associated to the release of fewer FFs.

It has been reported in previous studies^15,24^ that rotor spinning might be responsible for severe fiber damage and the higher generation and release of FFs as compared to ring and airjet spinning, mainly due to the aggressive opening action of the saw tooth opening roller during the yarn formation stage. This has been supplemented by the higher quantity of FFs released during the laboratory extraction process from rotor yarn samples. The FFs were extracted and quantified in those studies in yarn form rather than in fabric form. One study^15^ also quantified FFs by extracting them from dye effluent during yarn dyeing and pointed out the presence of a greater amount of FF in dye effluent as compared to the subsequent extractions in the laboratory. For example, 797 FFs/g (24.8 µg/g) were extracted from the dye effluent of rotor yarn as compared to 455 FFs/g (13.3 µg/g), 403 FFs/g (17.5 µg/g) and 215 FFs/g (8.5 µg/g) extracted during the first, second and third extraction cycles in the laboratory, respectively. This reinforced the hypothesis that a significant amount of FF is released to the dye effluent from the textile structure during wet processing (dyeing).^15^ The yarn is an intermediate textile product, which is woven or knitted and chemically processed afterwards under wet conditions. Pinlova et al.^24^ also proposed that already generated short FFs in yarns are released during subsequent wet processing, leading to an eventual decrease in the number and count of FFs during washing experiments. Therefore, in the current study, bespoke yarns were woven and dyed under the same controlled conditions before simulating the multiple washing cycles. Compared to the ring samples, the low release rate of FFs from rotor and airjet samples at the washing stage may be attributed to the release of some amount of pre-existing FFs in the fabric wet processing stage, where the textiles are chemically processed under wet conditions. The same reasoning may also be applied to the ring sample, where it may be anticipated that a certain number of relatively shorter length pre-existing FFs, generated during the spinning and weaving processes, were released during wet processing. It is known that fibers, yarns and fabrics are subjected to fiber–fiber and fiber–metal friction during different steps in textile manufacturing,^34^ which may cause damage to the textile fibers, resulting in the generation of FFs during textile manufacturing. Therefore, future research is inevitable to map the key process steps of the entire textile manufacturing processes, by collaborating between industry and academia, to better understand the role of each manufacturing step on the generation and/or release of FFs from textiles.

A few studies proposed that FFs might have been formed during the textile manufacturing processes and released from textile structures during subsequent laundering.^2,15^ Steel balls were used during the first to fifth wash cycles, in the present study, to accelerate the laundering process and fabric abrasion (to accelerate fiber damage) by agitating with the help of canisters and a lower liquor to fabric mass ratio. The hypothesis was that the steel balls would generate higher mechanical stresses during washing, leading to more abrasive friction and damage on fibers. However, it has been reported in a study that adding steel balls did not influence significantly the number and length of FFs released from textiles.^1^ Despite that, steel balls may induce mechanical stress on fibers and contribute to the generation of FFs during washing to some extent, depending on the mechanical properties of the fibers, but it is difficult to differentiate which FFs are produced during textile production and which are produced during laundering. Therefore, standardization of the extraction method of FFs, which could differentiate textile production induced FFs from those of laundering, is still a challenge for researchers.

### Characterization of fiber damage

**Figure 6.**
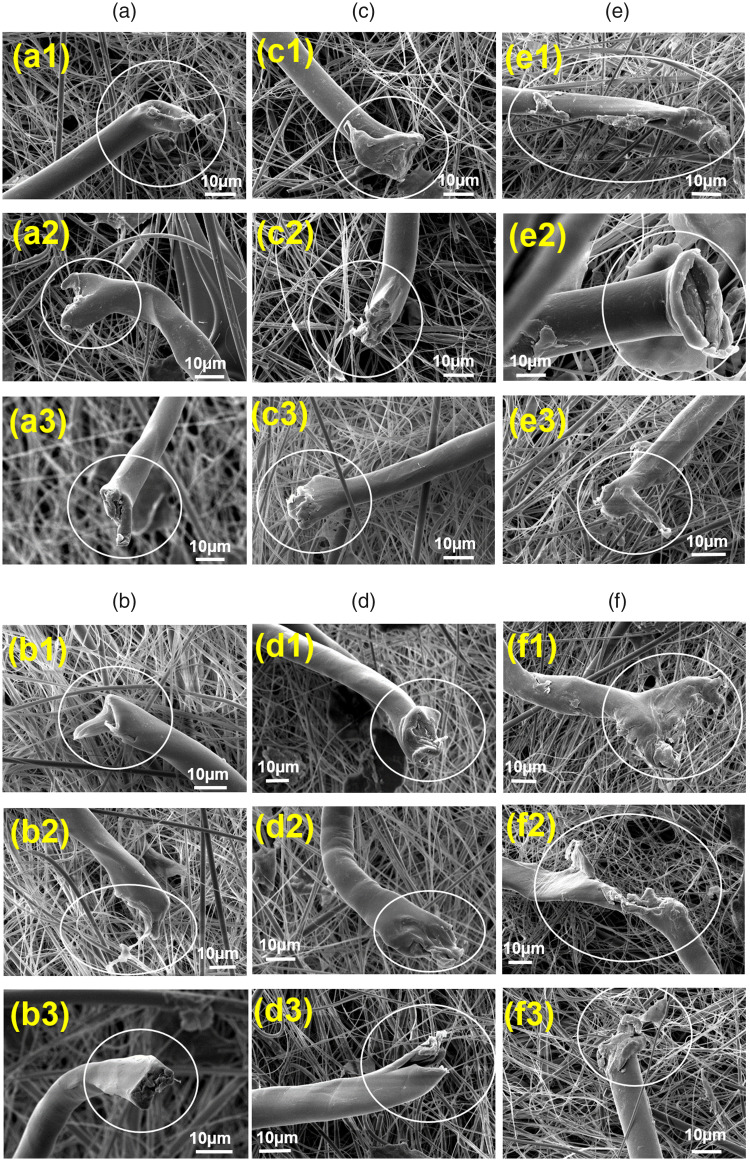
Characterization of damaged fiber ends released from samples: (a) ring pre-wash; (b) ring fifth wash; (c) airjet pre-wash and (d) airjet fifth wash; (e) rotor pre-wash and (f) rotor fifth wash.

**Figure 7.**
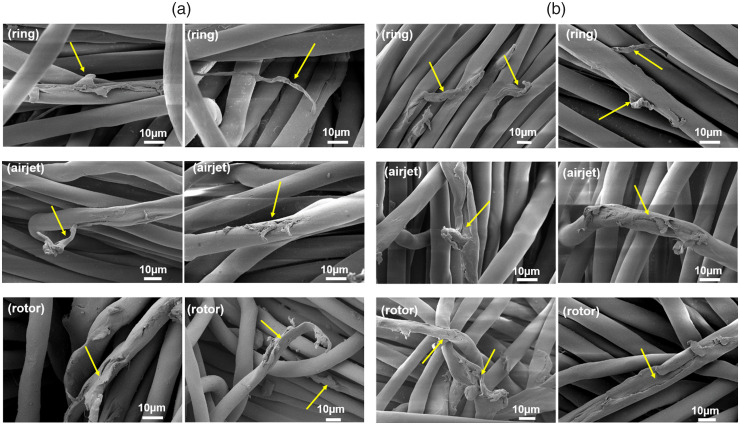
Scanning electron microscopy of fiber surface damage/wear observed on the surface of ring, airjet and rotor textile fabrics: (a) before washing and (b) after the fifth wash (color online only).

The damaged fiber ends of FFs released from ring, airjet and rotor samples after the pre-wash and fifth wash cycle were characterized using SEM to understand the mechanisms of damage on the basis of the details of broken fiber ends morphologies. The exposure of fibers to different types of stresses during textile processing can be associated with the nature of fiber damage and the exhibited morphology of observed fiber ends.^35^ The SEM images of damaged fiber ends are presented in Figure 6. The fiber end morphologies indicate solid damaged ends without significant axial fiber splitting. Based on the morphological shapes of the observed fiber ends from ring, airjet and rotor samples, the damaged ends are classified into perpendicular broken ends (Figures 6(a3), (b1) and (b3)), swollen or mushroom head broken ends (Figures 6(a2), (c1), (c3), (d1), (e2), (f1) and (f3)) and distorted or irregular shaped broken ends (Figures 6(a1), (b2), (c2), (d2), (d3), (e1), (e3) and (f2)). The damaged ends of FFs after the pre-wash and fifth wash do not show any particular change in their morphological shapes, which makes it difficult to identify the role of washing cycles in the generation of FFs and might suggest the presence of pre-existing fiber damage in the textile structure introduced during manufacturing, which is released during successive washing cycles. Apparently, the morphological details of damaged ends do not show the effect of differences in the yarn structure on the nature of fiber damage. Similarly, the fibrillation and surface damage of PET fibers was observed in all samples before washing and after the fifth wash, as highlighted by the yellow-colored arrows in Figure 7.

The morphologies of damaged fiber ends, identified by SEM analysis and shown in Figure 6, suggest that the most of damages are caused by high-energy loading conditions during the textile manufacturing. Almost similar findings were highlighted in previous studies,^1,24,36^ which suggested that these types of damages are potentially induced during the textile manufacturing processes. The textile manufacturing usually starts from processing densely packed bales of fibers. The fibers tufts are opened to the individual fiber level and parallelized before assembling them into a body of staple spun yarn. The staple yarns are converted into woven or knitted fabrics before wet processing, where the textiles are passed through dyeing and finishing processes. The manufacturing path of ring, airjet and rotor spun based textiles is comparable except that of rotor spinning, where the aligned fibers, in the form of slivers, are opened up again by an opening roller to reassemble them into the yarn body at the yarn formation stage. During the whole textile manufacturing chain, the textile fibers come in direct contact with high-speed moving machine parts and are also subjected to fiber–fiber and fiber–metal friction.^34^ It is estimated that a single fiber may have over 10 million contacts with metallic parts during staple yarn manufacturing.^37^ A recent study reported that FFs were present during each step of staple yarn production.^24^ Ishtiaque and Bhortakke^38^ classified broken PET fibers into direct breaks and indirect breaks based on their interaction with the wire points of the opening roller. They explained the mechanism of fiber breakage at the fiber opening stage of rotor spinning. If the proposed mechanism of fiber breakage in Ishtiaque and Bhortakke^38^ is considered valid, similar damage is likely to happen during the carding process where fibers are aggressively opened to the individual fiber level and bear high-energy deformation forces, which can contribute to the fiber damage and generation of FFs. Another study also identified fiber damage during the drafting stage of the ring frame.^39^ The same drafting principle is used during airjet spinning, which may also be responsible for fiber damage and the generation of FFs. Yarn hairiness is associated with greater release of FFs, as highlighted in previous studies,^31,32^ which favors the hypothesis that FFs are also generated in the subsequent processes after yarn manufacturing. Therefore, the role of subsequent processes, especially weaving, in the fiber damage and generation of FFs is also important where the yarns bear high-energy deformation forces during shedding and beating actions over the loom. However, no study is available in the literature to highlight the associated fiber damages, which should be carried out in the future to fill the knowledge gap.

The swollen or mushroom head type damage ends (Figures 6(a2), (c1), (c3), (d1), (e2), (f1) and (f3)) may result from high-speed tensile breaking from direct contact with metallic parts and localized heat generation and softening/flow of viscous material at the point of damage. However, different shapes of mushroom heads may be associated with the magnitude and direction of high-speed loading on the fiber during damage incidence. The perpendicular broken ends (Figures 6(a3), (b1) and (b3)) may be an outcome of direct damage to the fiber by a sharp contact point. During textile manufacturing, the fibers not only come in contact with metallic machine parts but are also entangled with each other, which may be considered responsible for fiber damage due to indirect loading. The distorted or irregular shaped broken ends (Figures 6(a1), (b2), (c2), (d2), (d3), (e1), (e3) and (f2)) may be the result of an indirect break from different loading conditions possibly due to entanglement of fibers with the neighboring fibers or by a combination of both direct and indirect contacts. A high-speed moving machine element may create a field of influence by indirect interaction with the neighboring fibers during fiber processing. In such scenarios, the fibers may experience different strain rates depending on the loading conditions, which may result in distorted or irregular shaped morphologies of damaged ends. Due to fiber–fiber and fiber–metal friction, the fibers experience surface abrasion during textile manufacturing,^34^ which may lead to fibrillation and surface wear of fibers, as highlighted in Figure 7(a). The same observation was reported in other studies.^24,36^ The surface damage of fibers observed in the washed samples may indicate some degree of abrasion due to the rubbing action of the fabric with the steel balls and walls of the canisters during washing. However, the limitation of this study is the difficulty in differentiating the production-induced surface damage from that of washing, which poses a challenge for future studies. However, a closer look at the samples after the fifth wash (Figure 7(b)) shows severe surface damage to fibers, which may be associated with damage during textile manufacturing.

## Conclusions

In the present study, ring, airjet (air vortex) and rotor yarns spun from 100% PET staple fibers were employed to develop dyed woven textiles. The textiles were subjected to repetitive accelerated laundering in the laboratory to identify the role of selected spun yarn structures on the release of FFs from textiles. The nature of fiber damage was studied by conducting SEM analysis of collected FF ends. It was found that airjet and rotor yarn structures had released significantly fewer FFs as compared to the ring yarn structure during the whole washing process. The length distribution data of FFs revealed the ring yarn structure to shed longer length FFs as compared to both the airjet and rotor ones. The fiber fractured ends highlight the significance of textile manufacturing processes to the generation of FFs. The swollen head, perpendicular broken and distorted broken shapes were identified as prominent types of fiber damages. The damage end morphologies do not show any apparent effect of differences in the yarn structure on the nature of fiber damage. The results of this study will be helpful to give a better understanding of the yarn structural effect of commercially acceptable technologies on the shedding of FFs.
